# A Liposomal Platform for Sensing of Extracellular Analytes Near Cells

**DOI:** 10.3390/bios8040117

**Published:** 2018-11-26

**Authors:** Xiaozhou Zhang, Sabrina Heng, Jinxin Pei, Jacqueline R. Morey, Christopher A. McDevitt, Andrew D. Abell

**Affiliations:** 1 ARC Centre of Excellence for Nanoscale BioPhotonics, Institute of Photonics and Advanced Sensing, Department of Chemistry, School of Physical Sciences, The University of Adelaide, Adelaide SA 5005, Australia; sabrina.heng@adelaide.edu.au (S.H.); jinxin.pei@adelaide.edu.au (J.P.); 2 Discipline of Physiology, Faculty of Health Sciences, The University of Adelaide, Adelaide SA 5005, Australia; 3 Research Centre for Infectious Diseases, School of Biological Sciences, University of Adelaide, Adelaide SA 5005, Australia; jmorey@illinois.edu (J.R.M.); christopher.mcdevitt@unimelb.edu.au (C.A.M.); 4 Department of Microbiology and Immunology, The Peter Doherty Institute for Infection and Immunity, University of Melbourne, Melbourne VIC 3010, Australia

**Keywords:** liposome, biosensing, extracellular sensing, fluorescence, small-molecule sensors

## Abstract

Cell-permeable fluorescent chemosensors (calcein, monochlorobimane, and a recently reported spiropyran-based sensor SP2) have been incorporated into yeast total lipid extract-based liposomes to suppress inherent cell permeability to allow the detection of extracellular Ca^2+^, GSH, and Zn^2+^, respectively. The repurposed sensors have enhanced aqueous solubility and the ability to quantitatively measure biologically relevant concentrations of Ca^2+^ (0.25 mM–1 mM), Zn^2+^ (6.25 µM–50 µM), and GSH (0.25 mM–1 mM) by fluorescence in aqueous media. In addition, the liposomal sensors are nontoxic to HEK293 cells and have the ability to detect exogenously added Zn^2+^ (1 mM), Ca^2+^ (1 mM), or GSH (1 mM) near cells without internalisation. This new sensing platform provides a means to repurpose a range of intracellular fluorescent sensors to specifically detect extracellular analytes, while also improving biocompatibility for overall enhanced use in a wide range of biomedical applications.

## 1. Introduction

The extracellular environment proximal to a cell membrane is critical to cellular function, directly influencing cell growth [1], differentiation [2], apoptosis [3], and migration [4]. A change in its composition can be indicative of disease, such as cancer [5,6,7], but is also associated with major physiological events, such as embryo fertilisation and development [8,9,10]. Of particular significance is the impact of extracellular metal ions and other analytes on embryonic health during early development [11,12]. Selective detection of these species has thus attracted considerable attention as a means to monitor embryo health in clinical applications, such as in vitro fertilisation (IVF) [13,14,15]. Small-molecule fluorescence-based sensors have found wide use in this context, providing high sensitivity and selectivity, as well as excellent spatiotemporal resolution in imaging studies [16,17,18]. However, these sensors generally act intracellularly [19,20,21]. This can be problematic, particularly in IVF applications where extracellular measurements are often required, because a sensor can be trapped within cumulus cells that surround the embryo, resulting in false positive or negative signals. There is, thus, a need for a simple, robust, and biocompatible approach to allow site-specific sensing of biologically important analytes, particularly in the extracellular environment proximal to the cell surface. Such a methodology should ideally be amenable to a range of chemosensors, and here we develop a new liposomal-based sensing platform as a general solution to this problem [22]. In particular, we show that a poorly soluble small-molecule fluorescent sensor embedded in a liposomal membrane retains its sensing capacity, while displaying enhanced aqueous solubility and diminished membrane permeability to allow specific extracellular sensing.

Extracellular Zn^2+^, Ca^2+^, and GSH were chosen as analytes for the study, as they are known to play a critical role in early embryonic development. For example, changes in concentrations of extracellular Ca^2+^, Zn^2+^, and GSH are associated with the fertilization of an embryo [23,24,25]. Measuring extracellular Ca^2+^, Zn^2+^, and GSH levels then provides an opportunity to identify the success, or otherwise, of fertilisation in clinical applications such as IVF. To address this need, three distinct fluorescent sensors (calcein, a recently reported [22] spiropyran-based sensor SP2, and monochlorobimane (mCB)) that detect these analytes were embedded into the membrane of liposomes constructed with yeast total lipid extract (denoted as LP). The ability of the resulting conjugates (LP–Cal, LP–SP2, and LP–mCB) to sense Zn^2+^, Ca^2+^, and GSH was then assessed. We demonstrate that embedding the sensor negates its ability to be internalised into HEK293 cells, allowing the sensing of Zn^2+^, Ca^2+^, and GSH in the extracellular matrix. This provides an efficient technique to repurpose intracellular fluorescent sensors for the specific detection of extracellular analytes for a wide range of biomedical applications.

## 2. Materials and Methods

### 2.1. General Information

All chemicals were purchased from Merck (Darmstadt, Germany) unless otherwise stated, and used without further purification. Calcein was purchased from TCI (Tokyo, Japan) and mCB from Sigma-Aldrich. SP2 was synthesised as previously described [22]. All lipids and extrusion apparatus were purchased from Avanti Polar Lipids (Alabaster, AL, USA) and fluorescence and absorption spectra were obtained using a Synergy H4 Microplate Reader unless otherwise indicated.

### 2.2. Determination of the Optimal Lipid Composition

The fluorescent sensor (calcein, SP2, or mCB) was dissolved in DMSO (20 µL, 8 mg/mL, 0.64% *w*/*w* to lipid) and mixed with a solution containing 25 mg of lipid (either 1,2-distearoyl-sn-glycero-3-phosphocholine (DSPC), 1,2-dioleoyl-sn-glycero-3-phosphocholine (DOPC), L-α-phosphatidylcholine from chicken eggs (egg PC), 1,2-dipalmitoyl-sn-glycero-3-phosphocholine (DPPC), or total lipids extracts from *Escherichia coli* or *Saccharomyces cerevisiae* (yeast) in 1 mL buffer (20 mM 3-(N-morpholino)propanesulfonic acid (MOPS), 5% maltose, pH 7.2)). Large multilamellar liposomes were formed by sonicating and vortexing the lipid-sensor mixture for 1 h. The mixture was clarified to remove unincorporated insoluble sensor by low speed centrifugation at 14,000 rpm for 20 min at room temperature, followed by ultracentrifugation at 50,000 rpm for 1 h at 25 °C to isolate the liposome fraction. The pellet containing liposomes was washed twice with a buffer (1 mL) to remove any unincorporated sensor and then resuspended in a buffer (1 mL). Unilamellar liposomes were obtained by extruding this mixture through a 0.4 µm membrane. After 11 passages, liposomes with a median size distribution of ~200 nm in diameter were generated for analysis. The fluorescence of all liposomal complexes (100 µL) in a buffer was then measured by a Synergy H4 microplate reader with λ_ex/em_ = 480/518 nm for all calcein complexes, λ_ex/em_ = 380/480 nm for all mCB complexes and λ_ex/em_ = 532/620 nm for all SP2 complexes. The mCB, calcein, and SP2 liposomal complexes were then separately mixed with GSH (1 mM), Ca^2+^ (1 mM) and Zn^2+^ (50 µM), respectively, and incubated in the dark for 10 min at room temperature. All concentrations of analytes reported are final concentrations of the solutions after mixing. The resultant fluorescence emission was similarly recorded. The experiments were carried out in duplicates. The fluorescence intensity of each sample was normalized to the fluorescence of the corresponding sample without analyte and plotted in GraphPad Prism 7.0.

### 2.3. Spectroscopic Characterization of LP–Cal, LP–mCB, and LP–SP2

LP–Cal, LP–SP2, and LP–mCB were similarly prepared using yeast total lipid extract and calcein, SP2 or mCB, respectively. This procedure was repeated three times for each liposome and the resulting mixture (100 µL) in an MOPS buffer was incubated for 10 min with or without analyte (1 mM Ca^2+^ for LP–Cal, 1 mM GSH for LP–mCB, and 50 µM Zn^2+^ for LP–SP2). The fluorescence of each sample was measured on the microplate reader and the intensity was normalised to the sample without analyte. This was then plotted using GraphPad Prism 7 as Figure S1 in Supporting Information. The liposomal complexes (100 μL) in a buffer (20 mM MOPS, 5% maltose, pH 7.2) were separately incubated with varying concentrations of Ca^2+^ (0–1 mM), Zn^2+^ (0–50 µM), and GSH (0–1 mM), respectively, for 10 min in the dark at room temperature. All analytes were first dissolved in water to make a concentrated stock solution. All concentrations of analytes reported are final concentrations of the solution after mixing. The resultant fluorescence spectra of LP–Cal (λ_ex_ = 480 nm), LP–mCB (λ_ex_ = 380 nm), and LP–SP2 (λ_ex_ = 532 nm) of each concentration of analyte were recorded on the plate reader. The experiments were carried out in duplicate in the dark. The fluorescence intensities at 518 nm for LP–Cal, 480 nm for LP–mCB, and 620 nm for LP–SP2 were plotted against analyte concentrations in µM, respectively, to produce a standard curve of calibration for each sensor. A linear trendline was fitted to the plots by GraphPad Prism 7.0. Blank liposomes without sensors were also prepared with yeast total lipid extract and their fluorescence in the presence and absence of analytes (Ca^2+^, Zn^2+^, and GSH) was similarly measured as a negative control. An averaged excitation spectrum (Figure S2A) was similarly obtained for a sample of LP–mCB (100 µM in an MOPS buffer), incubated with GSH (1 mM) for 10 min in the dark. This experiment was carried out in triplicate.

### 2.4. LIVE/DEAD Viability Assay

HEK293T cells were cultured in DMEM (Dulbecco’s Modified Eagle Medium) medium (Gibco, Grand Island, NY, USA) containing 10% heat inactivated fetal bovine serum (FBS) (Gibco)), 1% penicillin-streptomycin, 1% L-glutamine (MP Biomedicals, Santa Ana, CA, USA), and 2 ‰ fungizone (Thermo Fisher Scientific, Waltham, MA, USA). Cells were plated at 2.7 × 10^5^ cells/mL density in a 6-well plate. Cells were incubated in DMEM medium to reach 90% confluency. In the 75% MeOH group, cells were treated with 75% Methanol. 3 wells were treated with LP–Cal, LP–mCB, or LP–SP2 in PBS (Phosphate Buffered Saline), respectively, overnight. A LIVE/DEAD^®^ Viability/Cytotoxicity Kit (Molecular Probes, Eugene, OR, USA) was prepared with 1 µM calcein-AM and 10 µM ethidium homodimer-1. Cells were washed twice with warm PBS, then lifted and resuspended in an appropriate LIVE/DEAD^®^ Viability/Cytotoxicity assay buffer. In each well, 8 × 10^5^ cells were plated using a black walled 96-well plate. The plate was measured using a microplate reader.

### 2.5. Alamar Blue Viability Assay

Cell viability was quantified using the AlamarBlue assay (Molecular Probes, OR), as previously described [26]. The HEK293 cells were plated 8 × 10^5^ cells/well in 96-well plates, and the fluorescence signal levels were measured with a FLUOstar Optima microplate reader (BMG Labtech, Ortenberg, Germany) after 2 h or 24 h of incubation with LP–Cal, LP–mCB, or LP–SP2 in PBS to obtain quantitative measures of cell viability. Cells incubated in mercuric chloride (25 μM) were used as a negative control for cell viability and untreated cells were used as a positive control for cell viability.

### 2.6. Confocal Cell Imaging

HEK293T cells were cultured in DMEM medium (Gibco, USA) containing 10% heat inactivated fetal bovine serum (FBS; Gibco, USA), 1% penicillin-streptomycin (Sigma, USA), 1% L-glutamine (MP Biomedicals, USA), and 0.2% fungizone (Life Technologies, Australia). Cells were plated at 2.7 × 10^5^ cells/mL density in an 8-well ibidi μ-slide (ibidi, Berlin, Germany). Cells were incubated for 2 h in a PBS buffer containing 50 µL of LP–Cal, LP–SP2, or LP–mCB solution. For cell samples containing LP–Cal, LP–SP2, or LP–mCB, Ca^2+^ (1 mM), Zn^2+^ (1 mM), or GSH (1 mM) was added, respectively, and the samples were allowed to incubate for 30 min in the dark. The plate was then imaged on an Olympus FluroView V10i confocal microscope (Olympus, Tokyo, Japan) with excitation and emission wavelengths of 473 nm, 490–590 nm for LP–Cal; 405 nm, 420–520 nm for LP–mCB; and 559 nm, 570–670 nm for LP–SP2. Images were processed using ImageJ. Under “Brightness/Contrast” setting, the minimum level was set to 0 and the maximum level to 80 for all images. The fluorescence intensities reported in Figure S5 were also quantified in ImageJ. Using bright field image as a reference, six liposome-occupying areas of identical size were chosen randomly. Signal intensities were measured using the “Measure” function in ImageJ. Data were analysed in GraphPad and an unpaired t test was used within each liposome group with or without the addition of analyte.

## 3. Results and Discussion

### 3.1. Design and Preparation of Liposomal Sensors

**Figure 1:**
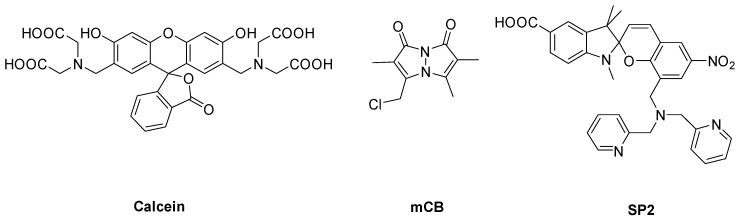
Structures of mCB, SP2, and calcein, selective sensors for GSH, Zn^2+^, and Ca^2+^, respectively. Calcein: λ_ex_ = 480 nm, λ_em_ = 518 nm; mCB: λ_ex_ = 380 nm, λ_em_ = 480 nm; SP2: λ_ex_ = 532 nm, λ_em_ = 620 nm.

Well-documented sensors for the detection of GSH and Ca^2+^, mCB, and calcein [27,28,29,30] were chosen for the liposomal studies, see Figure 1. A recently reported [22] red-emitting spiropyran-based sensor (labelled SP2, Figure 1) that selectively and reversibly detects Zn^2+^ was also used in order to expand the scope of the study. Calcein was selected due to its wide availability and common use with cells. Moreover, calcein is a member of the well-characterised fluorescein-based chemosensor family. Though the sensitivity of calcein to Ca^2+^ is pH dependent, it is capable to detect Ca^2+^ at a concentration used in all subsequent experiments at physiological pH (Figure S2B). Importantly, the three sensors encompass wavelength ranges commonly used in chemical sensing, with mCB emitting in the blue range (λ_em_ = 480 nm), calcein in the green range (λ_em_ = 518 nm), and SP2 in the red range (λ_em_ = 620 nm). All three sensors have been reported to function in biological environments to detect Ca^2+^, GSH, or Zn^2+^ by fluorescence [22,27,31,32,33]. Liposomal sensor conjugates for optimisation studies were prepared using DSPC, DOPC, egg PC, DPPC, and total lipid extracts from *E. coli* and yeast. These liposome–sensor complexes were assembled by mixing a DMSO solution of the sensor (8 mg/mL) with the lipid solution in an MOPS buffer (pH 7.2, 5% maltose) by sonication for 1 h at room temperature. The unincorporated sensors were subsequently removed by low-speed centrifugation (14,000 rpm) for 20 min. The resulting liposomes were isolated by high-speed ultracentrifugation (50,000 rpm) for 1 h. Unilamellar liposomes were then obtained by extrusion of this mixture through a 0.4 µm membrane. The size of the liposomes was measured by DLS (dynamic light scattering) with the thusly obtained Z-Ave and polydispersity index (PDI) reported in Table S1 of the supporting information.

**Figure 2:**
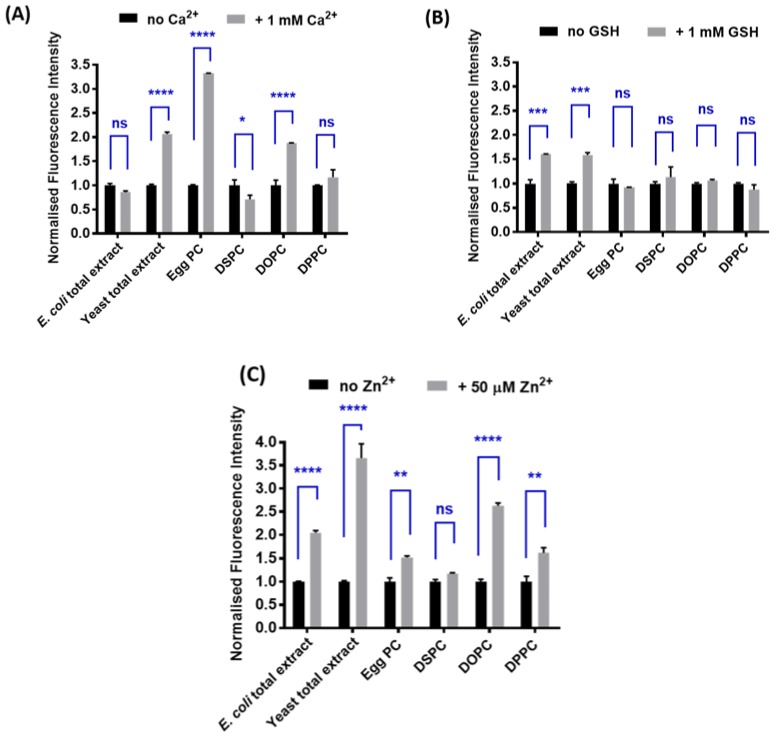
Normalised maximum fluorescence intensities of (**A**) liposomal–calcein complexes constructed with *Escherichia coli* total extract, yeast total extract, egg PC, DSPC, DOPC, or DPPC in the presence (grey bars) and absence (black bars) of Ca^2+^ (1 mM); (**B**) liposomal–mCB complexes constructed with *E. coli* total extract, yeast total extract, egg PC, DSPC, DOPC, or DPPC in the presence (grey bars) and absence (black bars) of GSH (1 mM); (**C**) liposomal–SP2 complexes constructed with *E. coli* total extract, yeast total extract, egg PC, DSPC, DOPC, or DPPC in the presence (grey bars) and absence (black bars) of Zn^2+^ (50 µM). All experiments were carried out in a 3-(N-morpholino)propanesulfonic acid (MOPS) buffer (20 mM, 5% maltose, pH 7.2) in duplicates. Error bars represent the SEM calculated from the duplicates. Unpaired t test was performed by GraphPad Prism 7.0 (shown in blue). * represents *p* ≤ 0.05, ** represents *p* ≤ 0.01, *** represents *p* ≤ 0.001, **** represents *p* ≤ 0.0001. Egg PC = L-α-phosphatidylcholine from chicken eggs, DSPC = 1,2-distearoyl-sn-glycero-3-phosphocholine, DOPC = 1,2-dioleoyl-sn-glycero-3-phosphocholine, DPPC = 1,2-dipalmitoyl-sn-glycero-3-phosphocholine.

The fluorescence of each liposome–sensor complex, both with and without added analyte (Ca^2+^ for calcein, Zn^2+^ for SP2 and GSH for mCB complexes), was measured in an MOPS buffer and the results are shown in Figure 2. Liposomes constructed from yeast total lipid extract provided the best sensing capability in all cases. All other liposomal constructs failed to retain sensing function for at least one of the sensors. For example, egg PC-derived liposomes are shown to provide the optimal sensing outcome for calcein (3-fold), but produce no fluorescence response for mCB. The yeast liposome–calcein complex (LP–Cal) and yeast liposome–SP2 complex (LP–SP2) demonstrate a >2-fold increase in fluorescence with added Ca^2+^ (1 mM) and Zn^2+^ (50 µM), respectively. A 1.6-fold increase was observed for the liposome–mCB complex (LP–mCB) with added GSH (1 mM). All liposomes derived from yeast total lipid extract, LP–Cal, LP–mCB, and LP–SP2, present as large unilamellar vesicles (LUV), with consistent sizes between 165–175 nm as determined by dynamic light scattering analysis (Table 1). The polydispersity index (PDI) for all preparations was less than 0.25, indicating that the liposomes had a relatively low level of polydispersity for a biological preparation [34]. Thus, liposomes formed from yeast lipid extract were selected for use in all subsequent studies based on their robust fluorescence response and consistent sizes. The preparation procedure for the yeast-derived liposomes was repeated three times, with a consistent sensing profile obtained for each repeat (See Figure S1, Supporting Information). This demonstrates the reproducibility of this approach.

### 3.2. Spectroscopic Characterization of Yeast Liposomal Sensors

**Figure 3:**
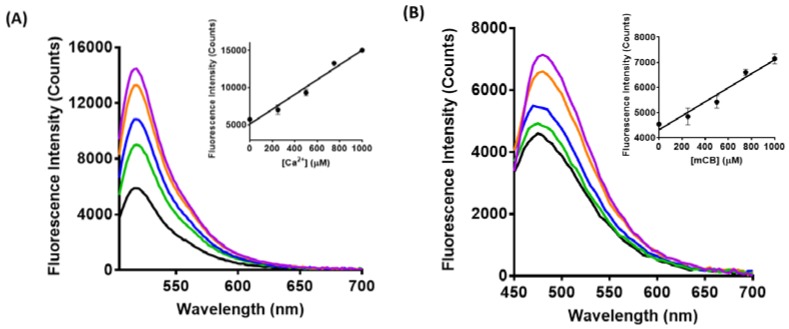

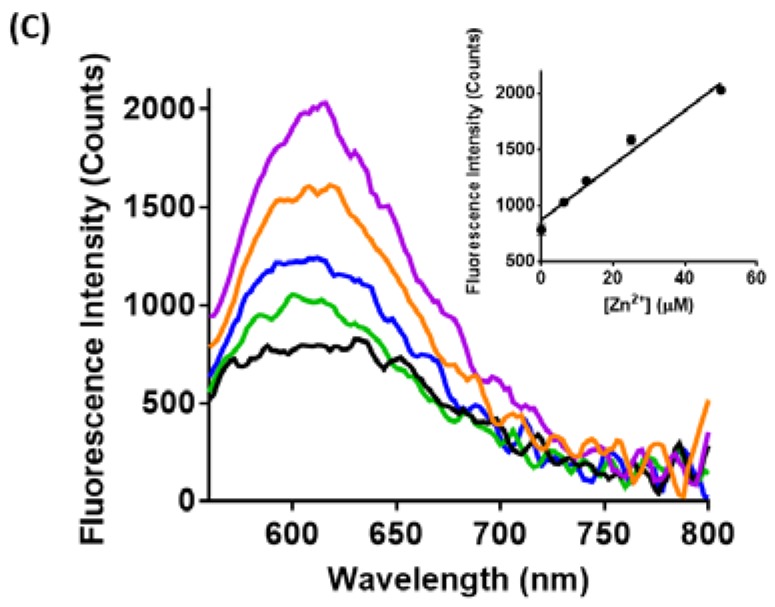
Fluorescence spectra of (**A**) LP–Cal (λ_ex_ = 480 nm) with no Ca^2+^ (black), 0.25 mM Ca^2+^ (green), 0.5 mM Ca^2+^ (blue), 0.75 mM Ca^2+^ (orange), 1 mM Ca^2+^ (purple); insert: Standard curve of calibration for LP–Cal in the presence of Ca^2+^, where fluorescence intensities at 518 nm were plotted against the concentrations of Ca^2+^ in µM (r^2^ = 0.9724). (**B**) LP–mCB (λ_ex_ = 380 nm) with no GSH (black), 0.25 mM GSH (green), 0.5 mM GSH (blue), 0.75 mM GSH (orange), 1 mM GSH (purple); insert: Standard curve of calibration for LP–-mCB in the presence of GSH, where fluorescence intensities at 480 nm were plotted against the concentrations of GSH in µM (r^2^ = 0.9604). (**C**) LP–SP2 (λ_ex_ = 532 nm) with no Zn^2+^ (black), 6.25 µM Zn^2+^ (green), 12.5 µM Zn^2+^ (blue), 25 µM Zn^2+^ (orange), 50 µM Zn^2+^ (purple); insert: Standard curve of calibration for LP–SP2 in the presence of Zn^2+^, where fluorescence intensities at 620 nm were plotted against the concentrations of Zn^2+^ in µM (r^2^ = 0.9746). All experiments were carried out in duplicates.

The fluorescence of LP–Cal, LP–mCB, and LP–SP2 in the presence of varying concentrations of Ca^2+^, Zn^2+^, and GSH, respectively, was determined to further characterise sensing capability in aqueous solution. Briefly, yeast-derived liposome samples (20 µL) were diluted with an MOPS buffer (70 µL) and 10 µL of a range of concentrations of Ca^2+^ (0–1 mM), GSH (0–1 mM), or Zn^2+^ (0–50 µM) were added. All concentrations reported are final concentrations. The mixtures were incubated for 10 min at room temperature in the dark. The resultant fluorescence was then measured using excitation wavelengths of 480 nm for calcein [35], 532 nm for SP2 [22], and 380 nm for mCB [36]. An increase in fluorescence intensity was observed upon addition of Ca^2+^, GSH, and Zn^2+^ to LP–Cal, LP–mCB, and LP–SP2, respectively (Figure 3), presumably due to the binding of the analyte to the corresponding sensors embedded in the liposome membrane, as reported previously [22,37]. The fluorescence of each yeast-derived liposomal complexes increased linearly with increasing analyte concentrations, as shown in the inserts of Figure 3. This is significant, as it indicates that the sensing capability of each separate sensor is retained on incorporation into the liposomal membrane. The limit of detection of LP–Cal, LP–mCB, and LP–SP2 is 0.25 mM (Ca^2+^), 0.25 mM (GSH), and 6.25 µM (Zn^2+^), respectively. By contrast, fluorescence was not observed for yeast-derived liposomes without an embedded sensor, with and without added analyte (see Figure S3, Supporting Information). Taken together, these data indicate that the observed fluorescence for LP–Cal, LP–mCB, and LP–SP2 upon addition of analyte arises from the embedded sensor. In addition, LP–Cal, LP–mCB, and LP–SP2 have consistent selectivity profiles to calcein [38], mCB [33], and SP [37], respectively (Figure S4, Supporting Information). Embedding poorly aqueous soluble mCB [33] and SP2 [22] into liposomes allows these sensors to be used in an organic solvent-free environment. Thus, incorporation of the sensors into a liposome enhances aqueous solubility and hence biocompatibility of the poorly soluble sensors, while retaining sensing function. This approach is applicable to a wide range of fluorescent sensors of low aqueous solubility to improve biocompatibility.

### 3.3. Imaging Ca^2+^, Zn^2+^, and GSH Near Live HEK293 Cells

**Figure 4:**
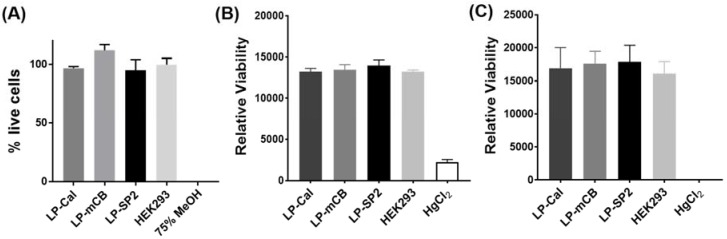
(**A**) The survival rate of HEK293T cells after incubation with LP–SP2, LP–mCB, and LP–Cal for 24 h. Each bar represents % of live HEK293 cells in each treatment group determined by normalising fluorescence intensity of calcein–AM cleaved by the cells measured with excitation and emission wavelengths of 490 nm and 510 nm to the sample without liposomes. The experiments were done in triplicates and the error bars represent the SEM calculated from the triplicates. (**B**,**C**) Viability assay of HEK293 cells incubated with the yeast-derived liposomal sensors LP–Cal, LP–mCB, and LP–SP2 for (**B**) 2 h; and (**C**) 24 h. Untreated HEK293 cells and HgCl_2_ treated cells were included as positive and negative controls.

**Figure 5:**
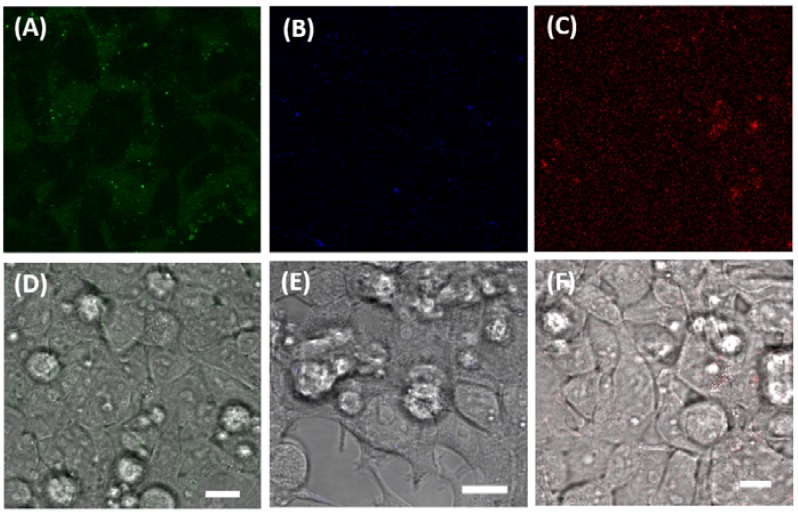
Confocal microscopic images of HEK293T cells (**A**) with addition of LP–Cal (50 µL) in the absence of Ca^2+^; (**B**) with addition of LP–mCB (50 µL) in the absence of GSH; (**C**) with addition of LP–SP2 (50 µL) in the absence of Zn^2+^. (**D**–**F**) represent overlays of (**A**–**C**) and the corresponding bright-field images. Excitation and emission wavelengths for (**A**) are 473 nm, 490–590 nm; for (**B**) are 405 nm, 420–520 nm; for (**C**) are 559 nm, 570–670 nm. Scale bars represent 10 µM.

Importantly, the incubation of each of the yeast-derived liposomal complexes with HEK293 cells over 24 h did not induce cytotoxicity relative to untreated cells, as evaluated by two separate viability assays, see Figure 4. HEK cells were also incubated for 2 h with LP–Cal, LP–mCB, or LP–SP2 in a PBS buffer (100 mM, pH 7.4) in the presence and absence of analytes (Ca^2+^ for LP–Cal, GSH for LP–mCB, and Zn^2+^ for LP–SP2; final concentration = 1 mM). The cells were then imaged on an Olympus FluroView V10i confocal microscope with excitation and emission wavelengths of λ_ex_/λ_em_ = 473/490–590 nm, λ_ex_/λ_em_ = 405/420–520 nm and λ_ex_/λ_em_ = 559/570–670 nm for LP–Cal, LP–mCB, and LP–SP2, respectively, in order to measure the fluorescence response of these complexes upon the addition of each analyte. Weak fluorescence was observed for any of the yeast-derived liposomal complexes in the absence of analyte, see Figure 5A–C. An overlay of the fluorescence and bright-field images (Figure 5) shows that all three yeast-derived liposomal complexes are not internalised by cells, but remain in close proximity to the cell surface. This observation is consistent with prior reports that liposomes smaller than 200 nm are less likely to be internalised into cells [39,40]. A significant increase in fluorescence intensity was measured at the emission wavelength corresponding to the liposomal sensor with the addition of analyte (see Figure S5, Supporting Information). Specifically, an 8-fold increase was observed for the GSH and LP–mCB treated cell sample compared to a sample without GSH, while a 5- and 7-fold increase was observed in similar experiments with LP–Cal and LP–SP2. These sensors are, thus, able to detect increasing concentrations of Ca^2+^, Zn^2+^, and GSH by fluorescence in a cell-based environment.

## 4. Conclusions

In summary, small molecule fluorescent sensors, calcein, mCB, and SP2, can be embedded into a liposomal membrane to improve biocompatibility, while retaining their ability to detect Ca^2+^, Zn^2+^, and GSH, respectively. These sensors are chemically distinctive and encompass emission wavelength ranges commonly used in sensing, with mCB, calcein, and SP2 representing the blue region (400 nm–480 nm), the green region (500 nm–550 nm), and the orange/red region (600 nm–700 nm), respectively. The nature of lipid used for the construction of the liposome is critical. Here, we established that yeast total lipid extract provided an optimal scaffold for all three sensors, as it generated liposomes of consistent size and retained the greatest level of fluorescence response upon exposure to analytes. Quantitative sensing of Ca^2+^ (0–1 mM), Zn^2+^ (0–50 µM), and GSH (0–1 mM) in aqueous solution was demonstrated using yeast liposome complexes, LP–Cal, LP–SP2, and LP–mCB, respectively. Further, we show the use of the yeast-derived liposomal sensors near HEK293 cells, without internalisation. Collectively, this work shows the repurposing of cell permeable sensors for extracellular sensing applications, negating the need for the synthesis and development of new sensor analogues.

## Figures and Tables

**Table 1:** Sizes of yeast liposome–SP2 complex (LP–SP2), yeast liposome–calcein complex (LP–Cal), and liposome–mCB complex (LP–mCB), measured by DLS.

Formulation	Z-Average ^1^ (d.nm)	PDI
LP–SP2	166 ± 2	0.21 ± 0.01
LP–Cal	165 ± 2	0.11 ± 0.04
LP–mCB	175 ± 1	0.19 ± 0.03

^1^ The values reported are the mean (± SEM) of 3 separate measurements.

## Supplementary Materials

The following are available online at http://www.mdpi.com/2079-6374/8/4/117/s1: Methods for dynamic light scattering measurements and selectivity assay, Table S1: Sizes of liposomes measured by DLS, Figure S1: Normalised fluorescence response of LP–Cal, LP–mCB, and LP–SP2 with and without added analytes, Figure S2: (A) Excitation spectrum of LP–mCB in the presence of 1 mM GSH, (B) Fluorescence of calcein with and without added Ca^2+^, Figure S3: Fluorescence emission of blank yeast total extract liposomes with and without analytes, Figure S4: Selectivity profiles of LP–Cal, LP–mCB, and LP–SP2, Figure S5: Fluorescence intensity of images of HEK293 cells treated with yeast-derived liposomal sensor with and without corresponding analyte.
