# Supplementary material for: A Liposomal Platform for Sensing of Extracellular Analytes Near Cells
Source: Biosensors (Basel). 2018 Nov 26;8(4):117. doi: 10.3390/bios8040117 (PMC6315562; doi:10.3390/bios8040117)
Supplement: Supplementary file 1 [file biosensors-08-00117-s001.pdf]

A Liposomal Platform for Sensing of Extracellular Analytes Near Cells

Xiaozhou Zhang [1,*], Sabrina Heng [1], Jinxin Pei [1,2], Jacqueline R. Morey [3], Christopher A. McDevitt [3,4] and Andrew D. Abell [1,*]

[1] ARC Centre of Excellence for Nanoscale BioPhotonics, Institute of Photonics and Advanced Sensing, Department of Chemistry, School of Physical Sciences, The University of Adelaide, Adelaide SA 5005, Australia; sabrina.heng@adelaide.edu.au (S.H.); jinxin.pei@adelaide.edu.au (J.P.)
[2] Discipline of Physiology, Faculty of Health Sciences, The University of Adelaide, Adelaide SA 5005, Australia
[3] Research Centre for Infectious Diseases, School of Biological Sciences, University of Adelaide, Adelaide SA 5005, Australia; jmorey@illinois.edu (J.R.M.); christopher.mcdevitt@unimelb.edu.au (C.A.M.)
[4] Department of Microbiology and Immunology, The Peter Doherty Institute for Infection and Immunity, University of Melbourne, Melbourne VIC 3010, Australia.
* Correspondence: xiao.z.zhang@adelaide.edu.au (X.Z.); andrew.abell@adelaide.edu.au (A.D.A.); Tel.: +61-8-8313-5652 (A.D.A.)

Received: 10 October 2018; Accepted: 21 November 2018; Published: 26 November 2018

Table S1. Sizes of liposomes measured by DLS.

Formulation	Z-Ave (d.nm)	PDI
E.coli-calcein	175	0.161
E.coli-mCB	220	0.295
E.coli-SP2	212	0.194
Egg PC- calcein	174	0.236
Egg PC- mCB	85	0.138
Egg PC- SP2	124	0.41
DSPC-calcein	361	1
DSPC-mCB	250	0.533

Formulation	Z-Ave (d.nm)	PDI
DSPC-SP2	250	1
DOPC-calcein	235	0.304
DOPC-mCB	287	0.477
DOPC-SP2	217	0.476
DPPC-calcein	79	0.078
DPPC-mCB	81	0.134
DPPC-SP2	79	0.056

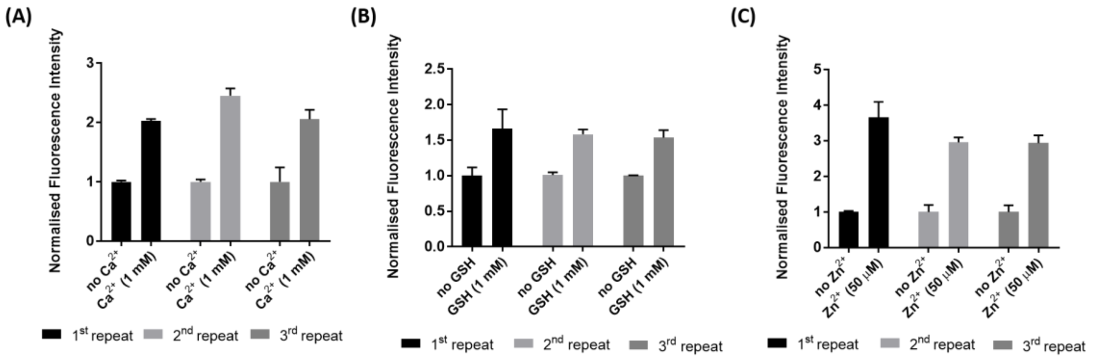

Figure S1. Normalised fluorescence response of (A) LP-Cal with and without added Ca^{2+} (1 mM); (B) LP-mCB with and without added GSH (1 mM); (C) LP-SP2 with and without added Zn^{2+} (50 μM). Each experiment was performed for three times in duplicates, with black bars representing data from first repeat, lighter grey bars representing data from second repeat and darker grey bars representing data from third repeat.

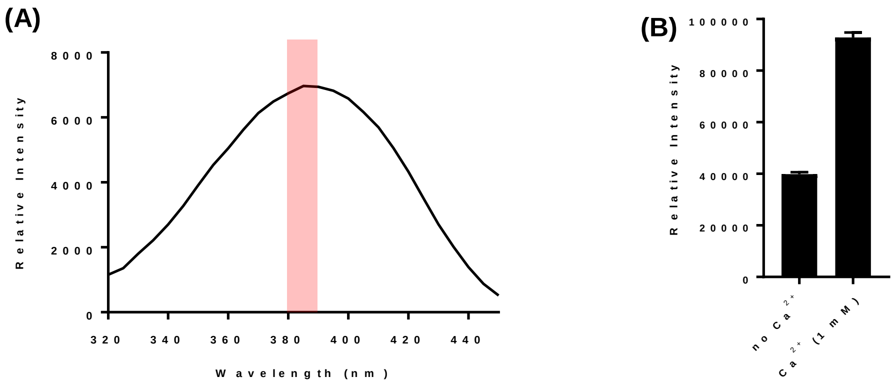

Figure S2. (A) Excitation spectrum of LP-mCB in the presence of 1 mM GSH. Region between 380 nm and 390 nm is highlighted in red. The spectrum is an average of a triplicate experiment. (B) Fluorescence of calcein (5 μM) in MOPS buffer with and without added Ca^{2+} (1 mM). The experiment was carried out in triplicate.

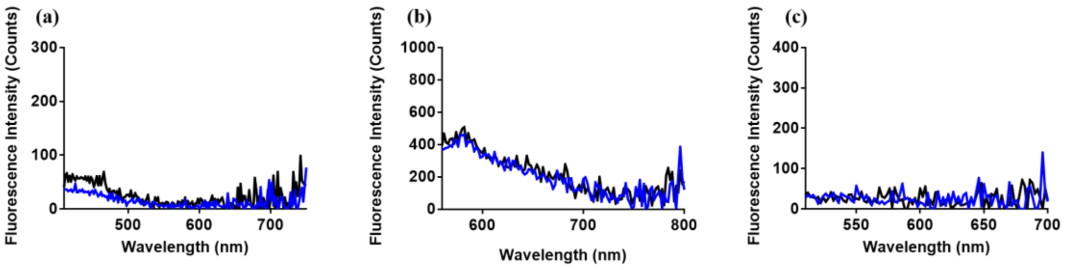

Figure S3. (a) Fluorescence emission of blank yeast total extract liposomes with (black) or without (blue) GSH (1 mM), λ_{ex} = 394 nm; (b) Fluorescence emission of blank yeast total extract liposomes with (black) or without (blue) Zn^{2+} (50 μM), λ_{ex} = 532 nm; (c) Fluorescence emission of blank yeast total extract liposomes with (black) or without (blue) Ca^{2+} (1 mM), λ_{ex} = 480 nm.

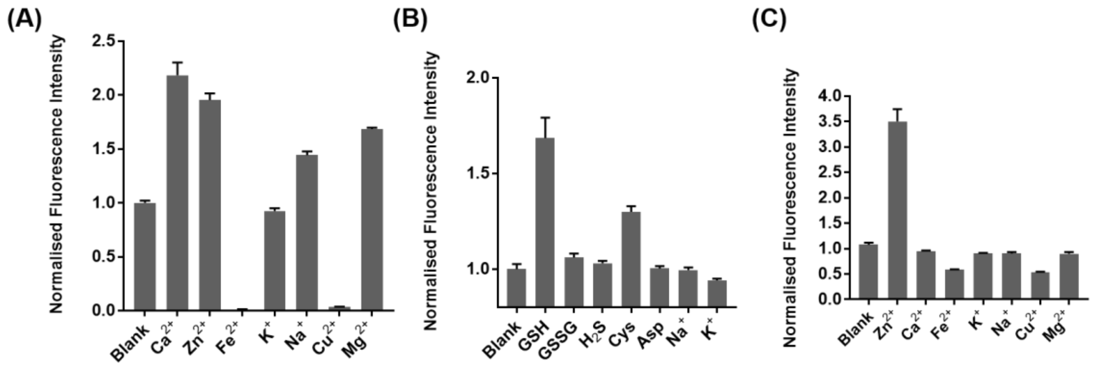

Figure S4. Normalised maximum fluorescence intensities of (A) LP-Cal in the presence of 1 mM Ca^{2+}, Zn^{2+}, Fe^{2+}, K^+, Na^+, Cu^{2+}, and Mg^{2+}; (B) LP-mCB in the presence of 1 mM GSH, GSSG, H_2S, Cys, Asp, Na^+, and K^+; (C) LP-SP2 in the presence of 50 μM Zn^{2+}, Ca^{2+}, Fe^{2+}, K^+, Na^+, Cu^{2+}, Mg^{2+}. All experiments were carried out in MOPS buffer (20 mM, 5% maltose, pH 7.2) in triplicates. Error bars represent the SEM calculated from the triplicates.

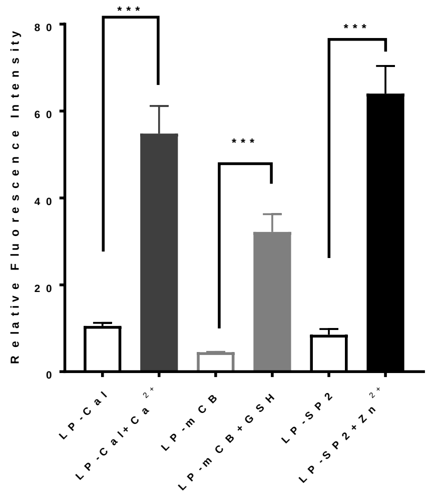

Figure S5. Fluorescence intensity of images of HEK293 cells treated with yeast-derived liposomal sensor with and without corresponding analyte. Six random liposome-occupying regions were chosen and their emission intensity was measured using ImageJ Fiji. Unpaired t test was performed using GraphPad 7 with *** representing $P \leqslant 0.001$.

Supplementary Experimental

Dynamic Light Scattering (DLS) Measurements. A liposome sample (40 μL) in buffer (20 mM MOPS, 5% maltose, pH 7.2) was placed in a low volume glass cuvette (ZEN2112, Malvern Instruments) and the specific light scattering intensity was measured by Malvern Nano ZS Zetasizer (Malvern Instruments, UK) using a 4 mV, 633 nm laser. Three measurements at angles of 13° and 173°, each with 12 scans, were collected for each liposome sample and the zeta average values were averaged to give the size of the particle. All experiments were done in triplicates.

Selectivity Assay. LP-Cal (100 μL) in buffer (20 mM MOPS, 5% maltose, pH 7.2) was separately incubated with Ca^{2+}, Zn^{2+}, Fe^{2+}, K^+, Na^+, Cu^{2+} and Mg^{2+} (1 mM) respectively for 10 min in the dark at room temperature. LP-mCB (100 μL) in buffer was similarly treated with GSH, GSSG, H_2S, Cys, Asp, Na^+, and K^+ (1mM) and LP-SP2 (100 μL) in buffer was similarly treated with Ca^{2+}, Zn^{2+}, Fe^{2+}, K^+, Na^+,

Cu^{2+} and Mg^{2+} (1 mM). All analytes were first dissolved in water to make a concentrated stock solution. All concentrations of analytes reported are final concentrations of the solution after mixing. The resultant fluorescence emission of each sample of LP-Cal (λ_{ex} = 485 nm), LP-mCB (λ_{ex} = 380 nm) and LP-SP2 (λ_{ex} = 532 nm) was recorded on the plate reader. The experiments were carried out in triplicate in the dark.

Fluorescence of Calcein at pH 7.2. Calcein (5 μM) was dissolved in buffer (20 mM MOPS, 5% maltose, pH 7.2) and placed in a 96-well plate. Ca^{2+} in the form of $Ca(ClO_4)_2$ was prepared as a stock solution of 100 mM in water. Ca^{2+} (final concentration = 1 mM) was added to the sample and incubated for 10 min in the dark. Fluorescence of the mixture was measured on a plate reader with λ_{ex} = 480 nm and λ_{em} = 518 nm.
